# The Not4 E3 Ligase and CCR4 Deadenylase Play Distinct Roles in Protein Quality Control

**DOI:** 10.1371/journal.pone.0086218

**Published:** 2014-01-17

**Authors:** David Halter, Martine A. Collart, Olesya O. Panasenko

**Affiliations:** Department of Microbiology and Molecular Medicine, Faculty of Medicine, Institute of Genetics and Genomics of Geneva, University of Geneva, Geneva, Switzerland; Boston University Medical School, United States of America

## Abstract

Eukaryotic cells control their proteome by regulating protein production and protein clearance. Protein production is determined to a large extent by mRNA levels, whereas protein degradation depends mostly upon the proteasome. Dysfunction of the proteasome leads to the accumulation of non-functional proteins that can aggregate, be toxic for the cell, and, in extreme cases, lead to cell death. mRNA levels are controlled by their rates of synthesis and degradation. Recent evidence indicates that these rates have oppositely co-evolved to ensure appropriate mRNA levels. This opposite co-evolution has been correlated with the mutations in the Ccr4-Not complex. Consistently, the deadenylation enzymes responsible for the rate-limiting step in eukaryotic mRNA degradation, Caf1 and Ccr4, are subunits of the Ccr4-Not complex. Another subunit of this complex is a RING E3 ligase, Not4. It is essential for cellular protein solubility and has been proposed to be involved in co-translational quality control. An open question has been whether this role of Not4 resides strictly in the regulation of the deadenylation module of the Ccr4-Not complex. However, Not4 is important for proper assembly of the proteasome, and the Ccr4-Not complex may have multiple functional modules that participate in protein quality control in different ways. In this work we studied how the functions of the Caf1/Ccr4 and Not4 modules are connected. We concluded that Not4 plays a role in protein quality control independently of the Ccr4 deadenylase, and that it is involved in clearance of aberrant proteins at least in part via the proteasome.

## Introduction

Messenger RNAs carry the information encoded within DNA to ribosomes where it is translated into the corresponding proteins. Errors regularly occur during protein synthesis. These errors may originate from the mRNA (mutations altering the coding sequence, secondary structure leading to ribosome stalling, etc…), resulting in production of defective proteins. To prevent the accumulation of such aberrant proteins cells have developed RNA quality control mechanisms that recognize defective mRNAs and degrade them efficiently [1], [2]. Errors also may occur at the protein level, when proteins fold inappropriately or interact with aberrant partners. This can be a consequence of aberrant mRNAs or due to unfavorable conditions such as lack of appropriate folding or assembly factors [3], [4].

mRNAs carry polyA tails that protect them from degradation and promotes translation in the cytoplasm. Removal of the polyA tail, deadenylation, is the first and rate-limiting step in mRNA degradation [5], [6]. In eukaryotic cells the Ccr4-Not complex provides the major deadenylation activity [7], [8], [9], [10] and thus it is an important player in RNA quality control. Besides mRNA degradation, this complex has been associated with other cellular activities, such as transcription and protein ubiquitination [11], [12], [13].

In the yeast *Saccharomyces cerevisiae* the Ccr4-Not complex is composed of nine core subunits, Not1-5, Caf1, Caf40, Caf130 and Ccr4. Not1 is the largest protein of the complex and the other subunits are organized around it. The Not2, 3 and 5 subunits form the Not module [14] and interact with the Not1 C-terminus [15], [16], [17]. Two ribonucleases, Caf1 and Ccr4, compose the deadenylation module [18] and bind a central domain of Not1 [17]. This structural organization is conserved in higher eukaryotes [19]. The Not4 subunit represents an E3 ligase module [20]. In yeast it is a stable subunit of the complex, but this is not the case of higher eukaryotes [21], [22], [23]. Nevertheless, the function of Not4 is certainly conserved, because the human protein complements the absence of the yeast protein [24].

Ccr4 and Caf1 are the subunits of the Ccr4-Not complex that compose the major eukaryotic deadenylase [25], [26], [27], [28]. They belong to 2 different types of deadenylation enzymes, Ccr4 - to the EEP-type family and Caf1 - to the DEDD-type family. In the yeast *S. cerevisiae* Caf1 contains a substitution in its catalytic site [29], [30]. Thus, only Ccr4 provides deadenylation activity *in vivo* and it is the primary yeast deadenylase. However in mammals and flies, Caf1 plays an important catalytic role in poly A tail shortening [8], [31], [32]. Caf1 bridges Ccr4 to Not1 [18] and this makes it essential for deadenylation activity *in vivo,* even in yeast [16].

Not4 is an E3 ligase of the RING family type [33] that catalyzes protein ubiquitination. The RING domain is located at the N-terminus of Not4 [34] and it is important for the ubiquitination activity of Not4, but not for its interaction with the Ccr4-Not complex [20], [35]. Several substrates of Not4 were described [36], amongst which are the ribosomal protein, Rps7A [37] and a ribosome-associated chaperone, NAC (the nascent polypeptide associated complex) [38], [39]. Consistently, Not4 and other subunits of the Ccr4-Not complex were found in translating ribosomes [37]. It was proposed that Not4 may ubiquitinate aborted proteins appearing during translational arrest and that this would lead to the degradation of these peptides by the proteasome [40]. Subsequent studies have indicated instead that the ubiquitination of aberrant products of translation mainly occurs via the E3 ligase Ltn1 [41]. Several other E3 ligases have been described to be involved in ubiquitination and degradation of misfolded proteins, such as Ubr1, Ubr2, San1 and others [42], [43], [44], [45].

A new role for Not4 in protein quality control was suggested by the recent finding that Not4 is involved in proteasome assembly [35], [36]. The proteasome is an important player in protein quality control [46], [47]. It is a large protease that eliminates aberrant proteins in the cell. It is composed of two main subcomplexes, the 20S core particle (CP) and the 19S regulatory particle (RP) [48], [49], [50]. RP is attached to one or both sides of the CP forming single- or double-capped proteasomes, respectively [51]. The RP is responsible for substrate recognition, deubiquitination and their translocation into the CP [52], [53], [54], whilst the CP provides substrate hydrolysis [55], [56], [57]. Appropriate RP and CP interaction and association into 26S proteasomes is important for normal proteasome function. Multiple factors are required for proteasome assembly and normal activity [58]. Not4 was shown to play a role in RP assembly that is important for normal RP-CP association [35].

Accumulation of polyubiquitinated proteins and increased aggregation was observed in the absence of Not4 [37]. To clarify whether that this is due to altered function of the deadenylation module of the Ccr4-Not complex in the absence of Not4, rather than to a problem with the proteasome, we have compared the involvement of the E3 ligase and deadenylase modules in protein quality control. We concluded that Not4 has a specific role in protein quality control that extends beyond regulation of deadenylation. This role consists, at least in part, in Not4’s importance for the functional integrity of the regulatory particle of the proteasome and might additionally include its role as an E3 ligase.

## Methods

### Cells

The *Saccharomyces cerevisiae* strains used in this work derive from MY1, BY4741 or SC0000 (Table 1). Single step deletions and gene tagging were performed by PCR. New strains were obtained from crosses. All media were standard.

**Table 1:** Yeast strains used in this study.

Strain	Genotype	Source
BY4741	*MATa his3Δ1 leu2Δ0 met15Δ0 ura3Δ0*	Euroscarf [80]
BY4742	*MATα his3Δ1 leu2Δ0 lys2Δ0 ura3Δ0*	Euroscarf [80]
SC0000	*MATa ade2 arg4 leu2-3,112 trp1-289 ura3-52*	Euroscarf [81]
MY1	*MATa ura3-52 trp1-1 gal2 leu2::PET56 gcn4Δ*	[82]
MY3417	Isogenic to BY4742 except *not4:: KanMX4*	Euroscarf
MY3419	Isogenic to BY4742 except *caf1:: KanMX4*	Euroscarf
MY3422	Isogenic to BY4742 except *ccr4:: KanMX4*	Euroscarf
MY3593	Isogenic to MY1 except *not4::KanMX4*	[38]
MY3621	Isogenic to MY1 except *caf1::TRP1*	[76]
MY4747	Isogenic to MY1 except *MATα ccr4::TRP1*	This work
MY4857	Isogenic to BY4741 except *not4::NOT4-TapTag-URA3*	[83]
MY5559	Isogenic to SC0000 except *rpn11::RPN11-TapTag-URA3*	[35]
MY5615	Isogenic to BY4741 except *MATα not4::HIS3*	[35]
MY7371	*MATα ade2 arg4 leu2-3,112 trp1-289 ura3-52 rpn11::RPN11-TapTag-URA3 caf1:: HIS3*	[35]
MY10004	*MATa ura3-52 leu2-Δ1 his3-Δ200 prc1-1 cim3-1*	From C. Mann [84]
MY10005	*MATa ura3 leu2-3,112 his3-11,15 pre1-1*	From D. Wolf [85]
MY10107	*MATα rpn11::RPN11-TapTag-URA3 ccr4::TRP1*	From the cross MY5559 x MY4747, this work
MY10402	Isogenic to BY4742 except *ubr1:: KanMX4*	Euroscarf
MY10404	Isogenic to BY4742 except *ubr2:: KanMX4*	Euroscarf
MY10406	Isogenic to BY4742 except *ltn1:: KanMX4*	Euroscarf
MY10408	Isogenic to BY4742 except *san1:: KanMX4*	Euroscarf

### Plasmids

A plasmid expressing CPY*-HA under control of the copper dependent promoter, *CUP1*, was a kind gift of T. Sommer [59]. Plasmids expressing GFP-K0-FLAG-HIS3, GFP-K12(AAA)-FLAG-HIS3, and GFP-R12-FLAG-HIS3, were a kind gift of T. Inada [40]. The plasmids expressing Not4_WT_, Not4_I64A_, and Not4*_ΔRING_* were described in [35].

### Yeast Growth Phenotypes

Yeast strains were grown to exponential phase, diluted to the same OD_600_ of 0.5. 5 µl of 10-fold serial dilutions were spotted on plates. Standard YPD or –URA media were used. Cycloheximide (CHX), hygromycin B (HygB) or azetidine-2-carboxylic acid (AZC) were added to the final concentrations of 0.05 µg/ml, 0.10 mg/ml or 0.10–0.50 mg/ml, respectively. Plates were incubated for several days at 30°C or, if indicated, at 37°C (heat sensitivity) or 16°C (cold sensitivity).

### Isolation of Aggregates

Isolation of aggregates was done as described in [37]. In order to analyze polyubiquitinated proteins, cell cultures were treated with *N*-ethylmaleimide (NEM) prior to harvesting as described in [60]. For this 10 mM of NEM was added to 50 ml of culture grown to an OD_600_ of 1.0. Cells were harvested and pellets were washed in 1 ml of cold water containing 10 mM of NEM and 10 mM of phenylmethylsulfonyl fluoride (PMSF). 10 mM of NEM was also added to all the buffers for aggregate isolation.

In order to analyze newly synthesized proteins in the aggregates the cell cultures were grown to an OD_600_ of 1.0. 50 ml of the cultures were harvested, washed 2 times with 20 ml of the media without methionine and incubated in 50 ml of this media for 1 h at 30°C under agitation. Cells were collected by centrifugation and resuspended in 5 ml of media without methionine. S^35^-labeled methionine was added at 20 µCi/ml in the media. Cells were incubated for 5 min at 30°C under agitation. 45 ml of cold water supplemented with 300 µg/ml of CHX was added to the cells. After 5 min incubation on ice cells were collected, washed with 1 ml of cold water with 300 µg/ml of CHX and frozen. After isolation of aggregates the samples were migrated on 4–12% gradient SDS gels and analyzed by laser scanner Typhoon FLA 7000 (GE Healthcare). The quantification was done with ImageQuant TL software (GE Healthcare).

### CPY* Stability

Cells transformed with the CPY*-HA plasmid [59] were grown exponentially in the presence of 0.1 mM of CuSO_4_. Cycloheximide was added to the cultures at OD_600_ of 0.6–1.0 at a final concentration of 200 µg/ml (+CHX). Control cultures were grown without cycloheximide (−CHX). Cells were collected at the indicated times. Samples were prepared by post alkaline lysis [61] and analyzed by western blot with an antibody against the HA tag, to follow CPY*-HA, and with an antibody against Egd2 for the loading control.

### Proteasome Activity

Proteasome activity in total extracts was analyzed as described in [62]. Cells were grown exponentially and 50 ml of culture were collected at OD_600_ of 1.0. Cell pellets were disrupted in the presence of 150 µl of lysis buffer (50 mM Tris-HCl, pH 8.0, 50 mM NaCl, 5 mM MgCl_2_, 1 mM EDTA, 10% glycerol, 1 mM ATP, 0.5 mM DTT) with 200 µl of glass beads for 15 min at 4°C. After spinning for 20 min at 16000 g the total protein concentration in the supernatant was adjusted to 15 mg/ml. 100 µg of total proteins were loaded on a 3.5% native gel prepared as in [62] and run for 3 h in a cold room. Gels were incubated with 100 µM of N-succinyl-Leu-Leu-Val-Tyr-7-amino-4-methylcoumarin (Suc-LLVY-AMC) for 20 min in the absence or presence of 0.02% SDS in order to detect latent proteasome activity. The activity was monitored at 365 nm.

### Proteasome Purification

Proteasome and proteasome subcomplexes purification was done from the strains expressing the Rpn11-ProteinA tagged subunit of the proteasome as described in [63].

### Ribosome Fractionation

Ribosomes were fractionated as in [37]. Briefly, 100 ml of yeast in exponential growth phase were treated with 100 µg/ml of CHX for 10 min on ice. Cells were harvested, washed with 50 ml of cold water with CHX, resuspended in 1 ml of buffer A (20 mM Hepes, pH8.0, 50 mM KCl, 10 mM MgCl_2_, 1% Triton X-100, 1 mM DTT, 1 mM PMSF, 100 µg/ml CHX, and protease inhibitor cocktail (Roche)) and pelleted. Cells were broken with 0.5 ml of glass beads in 0.5 ml of buffer A for 15 min at 4°C. The lysates were clarified by centrifugation at 14000 g for 10 min. 0.2 ml of lysates containing 3 mg of total protein was applied on a 12 ml 7–47% sucrose gradient in 20 mM Hepes, 50 mM KCl, 10 mM MgCl_2_, 100 µg/ml CHX and centrifuged for 150 min at 220000 g at 4°C. Fractions were collected using a UA/6 detector (ISCO, Inc.), precipitated with TCA and separated by SDS-PAGE.

### Antibodies

Anti-HA (anti-influenza hemagglutinin; Sigma) antibodies were used at the dilution 1∶5000. Anti-Egd2 antibodies (described previously [38]) were used at the dilution 1∶15000. Anti-ubiquitin antibodies (Biomol) were used at the dilution 1∶5000. Anti-Ssa1 and anti-Ssb1 antibodies were kindly provided by E. Craig and were used at the dilution 1∶15000. Anti-Rpt1 antibodies (Biomol) were used at the dilution 1∶10000. Anti- α1,2,3,5,6,7 (α1-7) antibodies (Biomol) were used at the dilution 1∶8000. Antibodies against Rpn8 were kindly provided by D. Finley and were used at the dilution 1∶10000. Anti-Rpl35 antibodies were kindly provided by M.Pool and were used at the dilution 1∶20000. PAP-antibodies (Peroxidise-anti-peroxidase soluble complex, Sigma) were used at the dilution 1∶10000. Anti-GFP antibodies (Roche) were used at the dilution 1∶5000.

## Results

### The Deletion of the Not4 E3 Ligase or the Ccr4/Caf1 Deadenylase have Different Phenotypes

**Figure 1:**
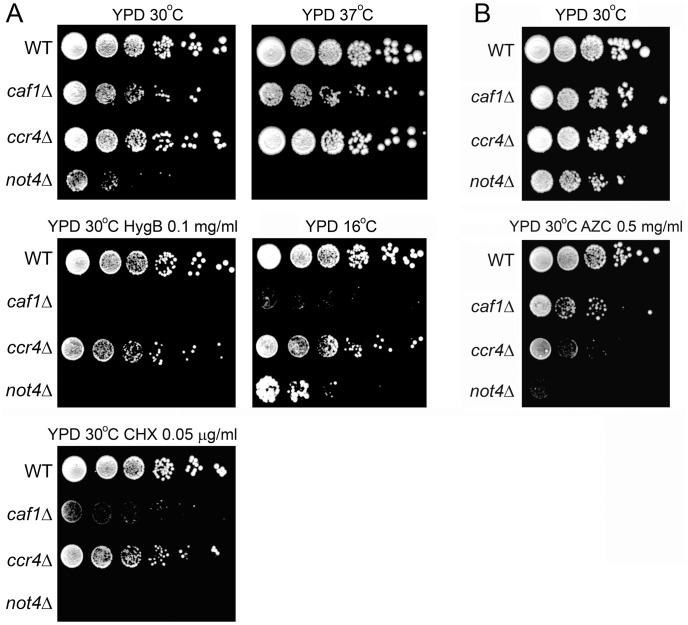
Deletions of the E3 ligase Not4, and the deadenylase subunits Ccr4 and Caf1, have different phenotypes. The indicated strains were grown to exponential phase and diluted to the same OD_600_ of 0.5. 10-fold serial dilutions were spotted on the YPD plates containing, when indicated, HygB 0.1 mg/ml; CHX 0.05 µg/ml; AZC 0.5 mg/ml, and left to grow for 4 days (A, except 16°C), for 17 days (A, 16°C) or for 6 days (B).

To determine to which extent the two enzymatic modules of the Ccr4-Not complex are functionally connected, we deleted the Not4 E3 ligase on one hand, and the subunits that play a role in deadenylation, either Caf1 or Ccr4, on the other hand. We compared growth of the wild type and mutants under different conditions: high and low temperature, and in the presence of agents affecting translation (cycloheximide (CHX), hygromycin B (HygB) or azetidine-2-carboxylic acid (AZC)) (Fig. 1). CHX inhibits translation and impairs proteasome function [64], [65]. HygB affects translational fidelity and increases read-through of stop codons [66]. AZC competes with proline during amino acid incorporation and induces misfolding of proteins, their degradation by the proteasome, and ribosome pausing [67], [68]. High temperature also affects translation since it leads to stalled ribosomes with translation arrested products [68], [69]. The Not4 deletion caused slow growth at 30°C and sensitivity to high temperature, CHX, HygB, and AZC. The deletion of Ccr4 lead to slight slow growth at 30°C but otherwise displayed no sensitivity or resistance to the conditions tested, except a slight sensitivity to AZC. Caf1 is necessary for the association of the Ccr4 deadenylase with the rest of the Ccr4-Not complex [18]. Its deletion had more severe phenotypes than the deletion of Ccr4. It reduced growth at 30°C on rich media and led to sensitivity to cold, HygB and CHX. Its impact on cell growth was less severe than the deletion of Not4: it did not lead to sensitivity to high temperature and was less sensitive to AZC. Interestingly, the deletion of Ccr4 or Caf1 led to reduced growth at 16°C, while deletion of Not4, in contrast, improved growth at low temperature (Fig. 1).

It was described that Not4 is involved in clearance of nascent chains upon translational arrest [40]. Several other E3 ligases were reported to have global roles in protein clearance: Ltn1 ubiquitinates nascent chains on the ribosome [41], [70], Ubr1 and Ubr2 play a role in degradation of misfolded cytosolic proteins [42] and San1 is involved in the proteasome-dependent degradation of aberrant nuclear proteins [43], [44]. The deletion of none of these other ligases had phenotypes similar to *not4Δ*, except for *ltn1Δ* that displayed decreased growth in the presence of HygB (Fig. S1).

### Not4 Deletion Causes Protein Aggregation in the Cell

**Figure 2:**
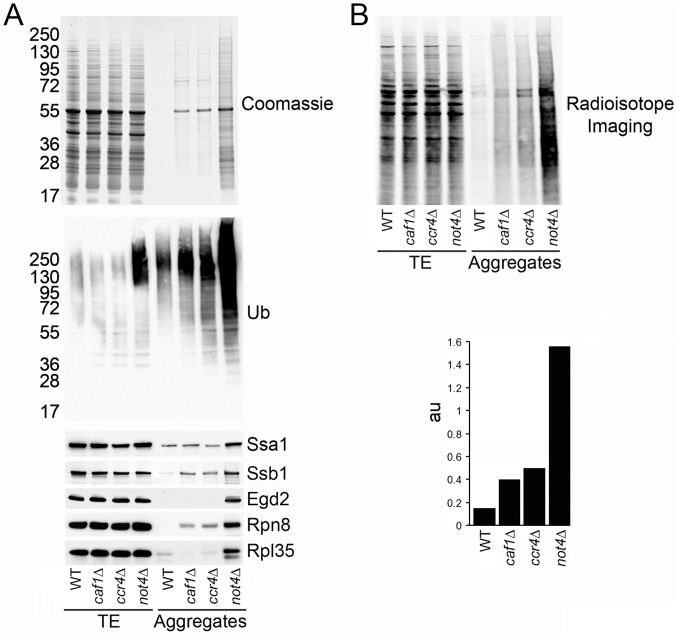
The Not4 deletion caused accumulation of aggregated and polyubiquitinated newly synthesized proteins. **A.** Aggregates were isolated from the indicated cells and analyzed by SDS-PAGE and Coomassie staining (upper panel), or western blot with antibodies against ubiquitin (middle panel), or against Ssa1, Ssb1, Egd2, Rpn8, and Rpl35 (lower panel). **B.** Aggregates were isolated from the same cells treated with S^35^-Met for 5 min and analyzed by SDS-PAGE and radioisotope imaging (upper panel). Images were quantified (lower panel). “au” is a ratio of the signal observed in the aggregates to the signal observed in the total protein fraction.

We have previously reported that the deletion of the several Not subunits of the Ccr4-Not complex (Not2, Not4 or Not5) caused increased protein aggregation in the cell [37]. Hence, we tested protein aggregation in cells in which the deadenylase module was deleted. We analyzed aggregates by SDS-PAGE and Coomassie staining (Fig. 2A, upper panel). The accumulation of protein aggregates in cells lacking Caf1 or Ccr4 was small and comparable to that in wild-type cells, whereas much stronger aggregation was observed in the *not4Δ* mutant.

We have also reported that polyubiquitinated proteins accumulate in *not4Δ* cell extracts [35]. So we compared the level of polyubiquitinated proteins in total extracts and in protein aggregates from wild-type and mutant cells lacking the enzymatic modules of the Ccr4-Not complex (Fig. 2A, middle panel). No increased level of polyubiquitinated proteins was detected in total extracts from *caf1Δ* or *ccr4Δ* cells compared to wild type. In contrast, in *not4Δ* cells polyubiquitinated proteins were observed in total extracts. A slight increase of polyubiquitinated proteins was observed in the aggregates from *caf1Δ* and *ccr4Δ* mutants, whereas very high levels of polyubiquitinated proteins were found in the aggregates from *not4Δ* cells. These aggregates in *not4Δ* contained the Hsp70 cytoplasmic chaperone, Ssa1; the ribosome associated chaperones Ssb1 and Egd2; the proteasomal protein, Rpn8; and the ribosomal protein, Rpl35 (Fig. 2A, lower panel).

To determine whether *de novo* synthesized proteins were contributing to the aggregates in the mutants, we did metabolic labeling of the cells with S^35^-methionine for 5 min. Aggregates were isolated from these cells and analyzed by radioisotope imaging (Fig. 2B, upper panel). Appearance of radioactive signal in the aggregates indicated that, indeed, newly synthesized peptides were aggregating, and this to a much greater extent in cells lacking Not4, than in cells lacking Caf1 or Ccr4 (Fig. 2B, lower panel).

Hence, loss of the ubiquitin ligase module of the Ccr4-Not complex provokes a severe accumulation of *de novo* synthesized and polyubiquitinated proteins. This cannot be accounted for simply by defective activity of the deadenylation module of the Ccr4-Not complex due to the absence of Not4. Indeed, the deletion of the deadenylation module of the Ccr4-Not complex does not by far have a comparable impact on accumulation of protein aggregates.

### Proteasome is Defective in *not4Δ*

**Figure 3:**
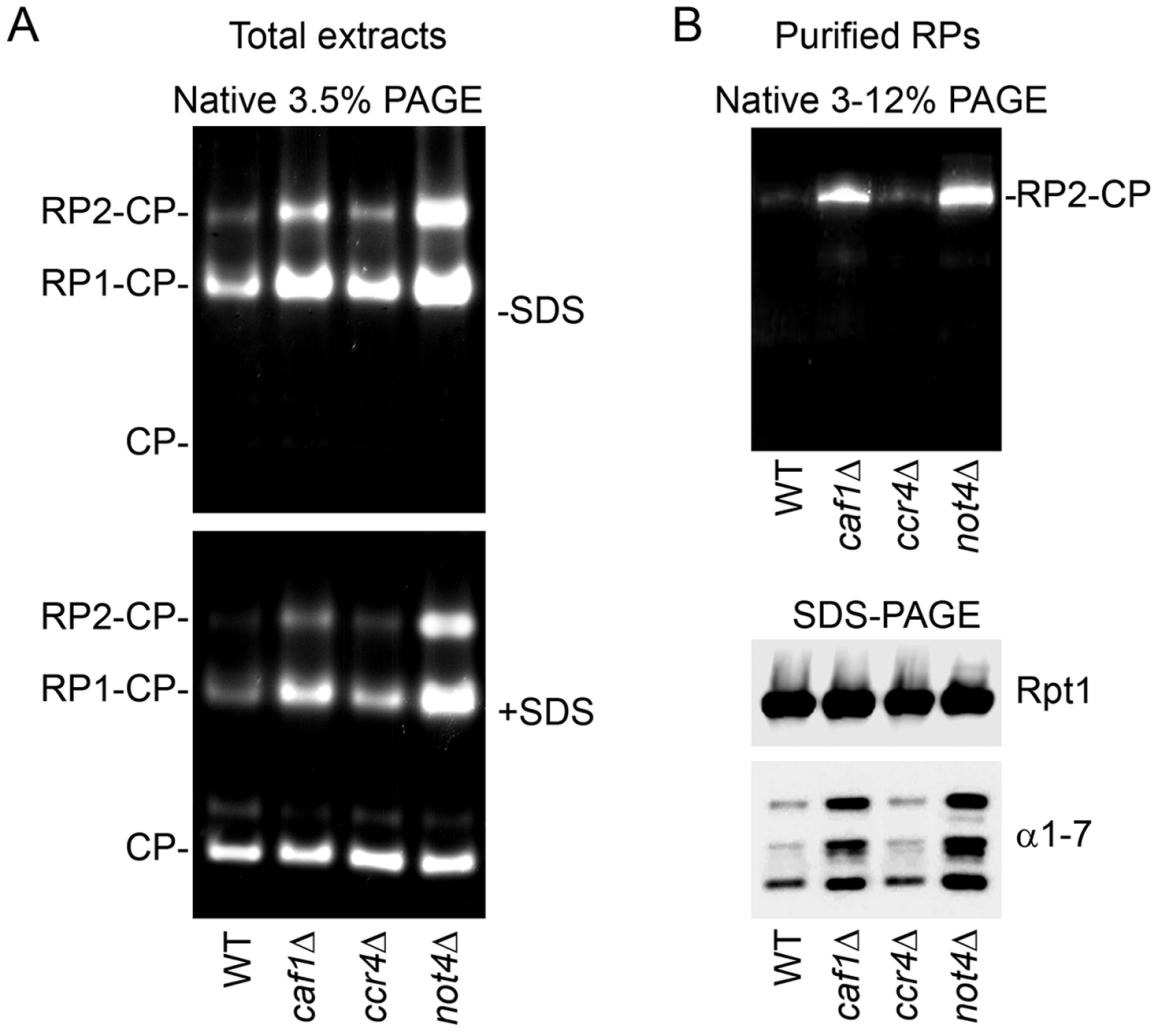
Proteasome was defective in *not4Δ* cells but not in *ccr4Δ* cells. **A.** Total cellular extracts were prepared from wild-type, *caf1Δ*, *ccr4Δ*, and *not4Δ* cells and loaded on 3.5% native gels. After electrophoresis gels were incubated with Suc-LLVY-AMC to analyze the proteasome activity in the absence (-SDS) and then in the presence (+SDS) of 0.02% SDS to detect the latent CP activity. The positions of double (RP_2_-CP) and single (RP_1_-CP) capped proteasomes and CP alone are indicated on the left. **B.** RPs were purified from wild-type, *caf1Δ*, *ccr4Δ*, and *not4Δ* cells, loaded on a gradient 3–12% native gel and then analyzed for activity (upper panel). The same purified material was analyzed by SDS-PAGE and western blot with antibodies against the RP subunit (Rpt1) and with antibodies against CP subunits (α1-7) (lower panel).

An important role of Not4 in functional assembly of the proteasome has been described [35], suggesting that accumulation of polyubiquitinated aggregated proteins in *not4Δ* might be partially due to their reduced clearance by the proteasome. Deletion of Not4 results in abnormal salt-resistant interaction between 2 proteasomal subcomplexes, regulatory particle (RP) and core particle (CP). This correlates with a greater level of proteasome activity measured with the substrate Suc-LLVY-AMC in extracts from *not4Δ* cell compared to wild-type cell extracts [35]. This observation was also true for some other mutants of the Ccr4-Not complex, in particular for *caf1Δ* (Fig. S6 in [35]). However, the proteasome has not been analyzed in the *ccr4Δ* mutant. Therefore we compared proteasomes isolated from cells deleted for Not4 or for Ccr4. We followed proteasome activity in the total extracts (Fig. 3A). As expected higher activities of double (RP_2_-CP) and single (RP_1_-CP) capped-proteasomes were detected in *caf1Δ* and *not4Δ* mutants. In contrast, activity of the proteasome from *ccr4Δ* was not significantly different than from the wild type, except for a very slight increase of RP-CP proteasome activity.

We also purified RPs from the different strains (Fig. 3B). The same amount of the RP subunit, Rpt1, was isolated from all strains (Fig. 3B, lower panel), indicating that the efficiency of the purification was identical. In wild-type cells the RP-CP interaction is salt-sensitive and incubation with high salt concentrations results in removal of CP subunits from RP. This is why no, or very little, amount of CP subunits (Fig. 3B, lower panel) and activity (Fig. 3B, upper panel) was detected in the purification of RP from wild-type cells under high salt. The same phenotype was observed for RP purified from the *ccr4Δ* mutant. In contrast, as we previously observed [35], salt-resistant RP-CP active complexes were purified via RP from *caf1Δ* and *not4Δ* mutants in high salt and CP subunits were detected (Fig. 3B, upper panel).

### Deletion of Not4 Stabilizes the Proteasomal Substrate, CPY*

**Figure 4:**
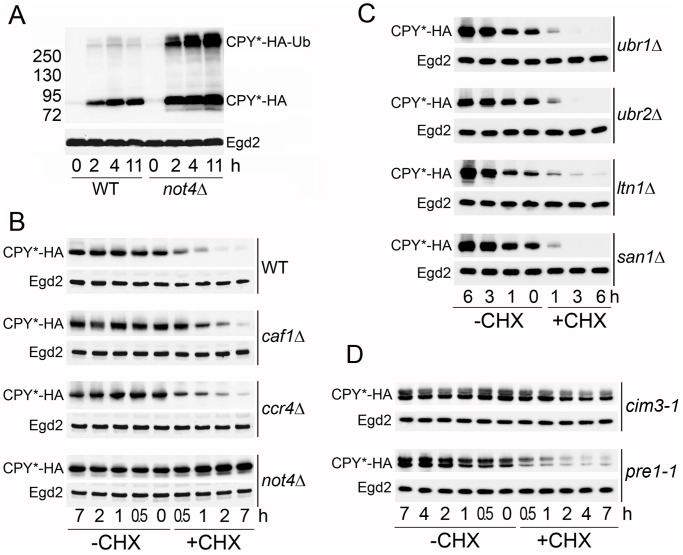
The deletion of Not4, but not the deletion of the deadenylase, stabilizes the proteasomal substrate CPY*. **A.** CPY*-HA was expressed from an episome under control of a copper dependent promoter in wild-type (WT) and *not4Δ* cells. Cells were exponentially grown without induction to OD_600_ of 0.6 (time 0). 0.1 mM CuSO_4_ was added to the media and cells were collected at indicated time points (2, 4 and 11 h) and analyzed by SDS-PAGE and western blot with antibodies against HA, to see CPY*-HA levels, and against Egd2 as a loading control. The positions of CPY*-HA and ubiquitinated CPY*-HA (CPY*-HA-Ub) are indicated on the right. The molecular weight markers are indicated on the left. **B.** Stability of CPY*-HA was analyzed in wild-type, *caf1Δ*, *ccr4Δ*, and *not4Δ* cells. Cells were grown exponentially and treated (+CHX) or not (−CHX) with CHX. Samples were collected at indicated time points and analyzed as in A. Since CPY*-HA expression was different in mutant strains (see Fig. S2A) 2 times less material was loaded on the gel in the case of *not4Δ* samples compared to wild type, and 4 times less material was loaded on the gel in the case of the *ccr4Δ* and *caf1Δ* samples compared to wild type. **C.** Stability of CPY*-HA was analyzed in *ubr1Δ*, *ubr2Δ*, *ltn1Δ*, and *san1Δ* cells as in B. **D.** Stability of CPY*-HA was analyzed in *cim3-1* and *pre1-1* cells as in B.

An increase in the proteasome activity with Suc-LLVY-AMC indicates that the interaction between RP and CP is not normal. The Suc-LLVY-AMC is a small artificial substrate that is not specifically targeted for proteasomal degradation like cellular protein substrates. It can be used for estimation of the peptidase activity of the proteasome *in vitro*. However, increased cleavage of Suc-LLVY-AMC *in vitro* does not directly reflect *in vivo* protease activity of the proteasome, which is complex and includes several steps (substrate recognition, deubiquitination, translocation to the CP, and, finally, cleavage). We decided to estimate protease activity of the proteasome in wild-type and mutant cells with an *in vivo* cellular substrate, CPY*. CPY* is a highly unstable mutated version of carboxypeptidase yscY (CPY), a lysosomal protein that is retarded in the ER lumen and rapidly degraded by the ubiquitin proteasome system [71], [72]. In our experiments we used an HA-tagged version of CPY* expressed under control of the copper dependent promoter, *CUP1*. In wild-type and *not4Δ* cells, CPY* was well induced after 2 h of copper treatment, and the level was not dramatically changed after longer induction times (Fig. 4A). So for the rest of the experiments we grew the cells in the constant presence of copper in the media. In both wild-type and *not4Δ* strains, we detected slower migrating forms of CPY*, that correspond to ubiquitinated CPY*. The level of CPY* was higher in *not4Δ* compared to wild type, but, more importantly, the level of ubiquitinated CPY* was much greater in *not4Δ*.

We then compared CPY* stability in wild-type, *caf1Δ*, *ccr4Δ*, and *not4Δ* cells (Fig. 4B). In wild-type cells the level of CPY* was noticeably reduced after 30 min of incubation with CHX, consistent with a described half-life of CPY* of about 24 min [71]. CPY* was similarly unstable in *caf1Δ* and *ccr4Δ* mutants (Fig. 4B). Surprisingly, CPY* was expressed at very high levels in *caf1Δ* and *ccr4Δ* mutants (Fig. S2A). This increase did not correlate with any comparable increase of the mRNA levels (Fig. S2B) suggesting that, instead, translation of CPY* may be higher in these mutants, since Caf1 and Ccr4 have been associated not only with mRNA deadenylation but also translational repression (reviewed in [13]). CPY* was strongly stabilized when Not4 was deleted. Even after 24 h of protein synthesis arrest, the level of CPY* in *not4Δ* was not reduced (data not shown). We also tested CPY* stability in cells mutated for the Ubr1, Ubr2, Ltn1, and San1 E3 ligases (Fig. 4C). In all of these mutants CPY* was as unstable as in wild-type cells, indicating that, if these quality control E3 ligases participate in CPY* degradation, they are redundant, while Not4 might have a global role in clearance of CPY*, probably acting via the proteasome.

Because of the role of Not4 in the functional assembly of the proteasome, we considered that the stability of CPY* in *not4Δ* could be due to altered proteasome function. Hence, we tested the stability of CPY* in two proteasome mutants, *cim3-1* and *pre1-1*, mutant alleles of genes encoding the Rpt6 RP subunit and the β4 CP subunit of the proteasome, respectively. In both mutants proteasome was not active for cleavage of Suc-LLVY-AMC (data not shown). CPY* was stable in the *cim3-1* mutant, while in the *pre1-1* mutant it was unstable (Fig. 4D). These results indicate that different proteasome mutants differently affect stability of CPY*, as previously observed [73], [74], and, in particular, that functional integrity of RP is required for degradation of CPY*. In this context it is important to underline that cells lacking Not4 displayed 2 distinguishable defects in proteasome integrity: besides carrying salt-resistant RP-CP proteasomes, a phenotype shared by *caf1Δ* as shown above (Fig. 3), it also led to unstable free RP [35]. Hence, our current findings that CPY* is stabilized in *cim3-1* as in *not4Δ* lead us to conclude that cells lacking Not4 fail to degrade CPY* because of defective RP.

### Use of Not4 Mutants to Characterize the Role of Not4 in Protein Quality Control

**Figure 5:**
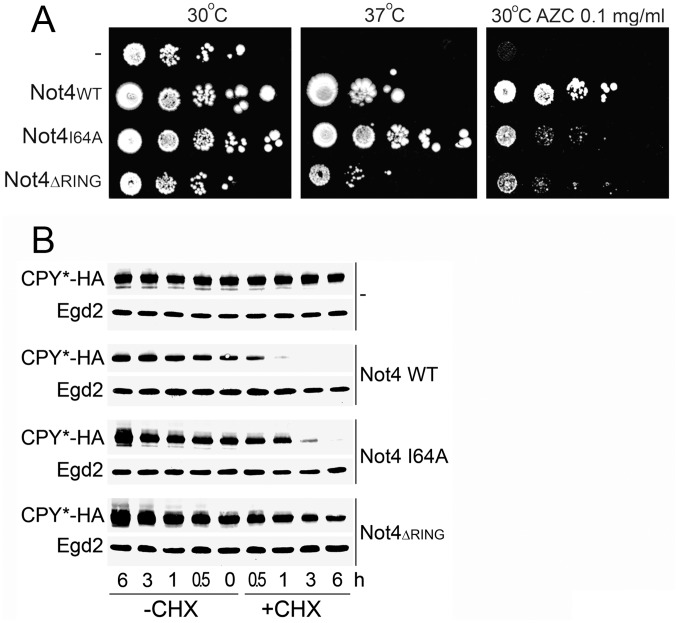
Use of Not4 mutants to characterize the role of Not4 in protein quality control. **A.** *not4Δ* cells expressing only vector (−) or vector containing the Not4 derivatives (Not4_WT_, Not4_I64A_ or Not4*_ΔRING_*) were analyzed as in Fig. 1 and spotted on – URA plates and left to grow for 13 days at 30°C or 37°C. When indicated, plates contained 0.1 mg/ml of AZC. **B.** Stability of CPY*-HA was analyzed in *not4* mutants as in Fig. 4B.

Not4 has been suggested to play a role in co-translational protein quality control by acting directly as an E3 ligase for ubiquitination of translationally-arrested proteins [40]. Not4 could also be important for protein quality control because of its importance for functional integrity of the proteasome [35] that clears aberrant proteins. We studied Not4 mutants to define the relevance of these 2 phenotypes for growth on media affecting translation, namely high temperature and AZC. The RING domain of Not4 is important for substrate ubiquitination and proteasome integrity [35], [37]. In contrast, a point mutation within the RING domain of Not4 (I64A) affects the interaction of Not4 with E2 partners [75] and reduces the ubiquitination of its Egd2 substrate (Fig. S3A in [35]), but it does not influence proteasome integrity [35]. We analyzed the growth phenotypes of either the Not4 I64A point mutant or a mutant bearing an entire deletion of the RING domain (Not4*_ΔRING_*) (Fig. 5A). The Not4*_ΔRING_* was very sick but distinguishable from the null mutant: it grew better than *not4Δ* at 37°C and in the presence of AZC. However, CPY* was similarly stabilized in both Not4*_ΔRING_* and in the null mutant (Fig. 5B). The I64A mutant grew slightly slower at 37°C and in the presence of AZC compared to the wild type, and CPY* was slightly stabilized (Fig. 5A and B). The I64A mutant was also sensitive to CHX [35] and HygB [75].

These results indicate that Not4 is likely to play a role in protein quality control that extends beyond its impact on the proteasome and involves its function as an E3 ligase.

### Not4 Accumulates in Polysomes in Response to AZC and High Temperature

**Figure 6:**
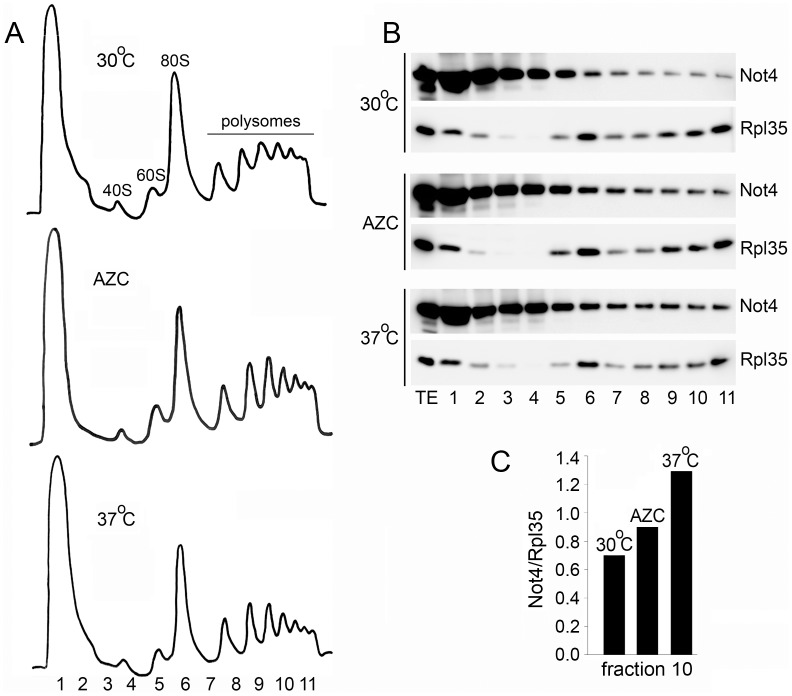
Not4 accumulates in polysomes in response to AZC and high temperature. **A.** Polysome profiles from the cells expressing Not4-ProteinA. Cells were exponentially grown on YPD media at 30°C or 37°C, as indicated, and collected at OD_600_ of 1.0. When indicated, cells were treated with 0.4 mg/ml of AZC. AZC was added at OD_600_ of 0.15 and cells were grown till OD_600_ of 1.0 and collected. Extracts, containing 3 mg of total proteins, were subjected to 7–47% sucrose gradient centrifugation and analyzed by UV reading at 254 nm. Fraction numbers and the positions of 40S, 60S, 80S, and polysomes are indicated. **B.** Fractions were collected and analyzed by western blot with PAP and Rpl35 antibodies. **C.** Not4 content in polysomes was quantified. For this the Not4 signal in polysomes (fraction 10) was quantified with ImageQuant TL software (GE Healthcare) and normalized on the Rpl35 signal.

Not4 is important for temperature and AZC resistance (see above, Fig. 1), it is present in polysomes [37], [40] and it is thought to ubiquitinate translationally-arrested protein products [40]. To clarify better the role of Not4 in protein quality control, we analyzed what happens with Not4 under conditions that adversely affect protein synthesis. We analyzed ribosome profiles from cells grown in the presence of AZC and high temperature. Not4 accumulated in polysome fractions upon incubation of cells with AZC and, particularly, upon incubation of cells at high temperature (Fig. 6). These results are consistent with the idea that the presence of Not4 on actively translating ribosomes is important under these conditions.

**Figure 7:**
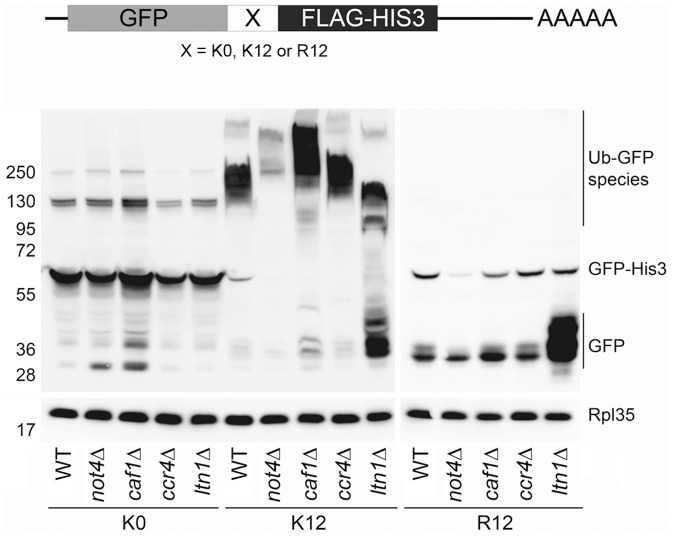
Role of the Not4 and Caf1/Ccr4 modules in co-translational degradation. Indicated strains were transformed with GFP-X-FLAG-HIS3 plasmids, that either contains no positively charged stretch between GFP and FLAG-HIS3 moieties (K0), or contains 12 lysine residues (K12), or 12 arginine residues (R12) [40]. Cells were grown to exponential phase, collected and analyzed by post alkaline lysis. The same amount of the samples were loaded on 4–12% SDS gels and after electrophoresis analyzed by western blot with antibodies against GFP and against Rpl35 as a loading control. Positions of GFP arrested products, full length GFP-X-FLAG-HIS3 products and GFP containing ubiquitinated species are indicated on the right.

We then investigated how Not4 and the deadenylase subunits Ccr4 and Caf1 might be important for dealing with translationally-arrested products. We transformed wild-type, *not4Δ*, *ltn1Δ*, *caf1Δ*, and *ccr4Δ* cells with constructs described in [40]. In these constructs GFP fused to FLAG-HIS3 is expressed without (K0), or with a positively charged stretch of 12 lysines (K12) or 12 arginines (R12) inserted between GFP and the FLAG-HIS3 moieties. With these constructs it was shown that translationally-arrested products accumulated in *not4Δ* cells, when a positively charged stretch of amino acids was present in the middle of the open reading frame (ORF) [40]. We observed that relatively equal levels of full length GFP-K0-HIS3 product were detected in all strains (Fig. 7). Full length GFP-R12-HIS3 was also detectable for all strains, but it was less abundant compared to the levels of GFP-K0-HIS3, and it was particularly lower in *not4Δ* than in the other cells. In all R12 transformants translationally-arrested GFP (described in [40]) was additionally detected. The levels of this arrested product were relatively equal for all strains except for *ltn1Δ*, where they were dramatically increased. This observation is compatible with the previous reports indicating that Ltn1 is responsible for ubiquitination and degradation of translationally-arrested proteins [41], though it is surprising that no ubiquitinated forms of GFP from the R12 transformants were detected in any of the strains.

The levels of full length GFP-K12-HIS3 were significantly reduced compared to GFP-K0-HIS3 or GFP-R12-HIS3. A very low level of translationally-arrested GFP was detected in the wild type and even less in *not4Δ*. Slightly more of arrested GFP was detected in *caf1Δ.* Significantly greater amounts of this product were detected in *ltn1Δ* cells, an observation consistent with the role of Ltn1 in degradation of translationally-arrested proteins [41]. Interestingly, in the case of these K12 constructs, many high molecular weight GFP species were detected in all strains. They probably correspond to ubiquitinated derivatives (Fig. 7, Ub-GFP). The migration of the Ub-GFP smear was similar in wild-type and *ccr4Δ* cells, but it migrated slower in *not4Δ* and *caf1Δ.* This correlates with altered proteasome integrity in these 2 mutants, and may indicate that the mostly highly polyubiquitinated forms of GFP do not get deubiquitinated and degraded by the proteasome in these mutants. In contrast, the smear was smaller and faster migrating in *ltn1Δ* cells, in good correlation with the role of Ltn1 in ubiquitination of translationally-arrested proteins. Clearly, however, Ltn1 cannot be the sole E3 enzyme involved, since much residual ubiquitination is observed in the absence of Ltn1. It is noticeable that the amount of these Ub-GFP forms was significantly reduced in *not4Δ.* In fact, the detectable total amount of protein produced from the K12 construct in *not4Δ* was reduced compared to the wild type. Total levels of protein detected from the R12 construct were also reduced in *not4Δ* compared to the wild type, whereas the amount of protein produced from K0 was similar in wild type and *not4Δ*. This indicates a specific role of Not4 for preserving translation, and stability of mRNA or protein from constructs that lead to translational arrest in the middle of the ORF. No such role could be observed for Ccr4, and in this context it is important to note that while polysomes were reduced in *not4Δ* [14], no such reduction was observed in *ccr4Δ* (Fig. S3).

## Discussion

In this report we compared the function of the 2 enzymes of the Ccr4-Not complex, the Ccr4 deadenylase, and the Not4 E3 ligase. We also analyzed the Caf1 subunit of the complex, which does not have deadenylation activity in baker’s yeast, but is important for the connection of the Ccr4 deadenylase with the rest of the Ccr4-Not complex and, therefore, is important for the deadenylation activity of Ccr4. We came to the conclusion, that the function of Not4 in protein quality control is separable from the role of the deadenylase, and it is specific: 1) the Ccr4 deletion led to different growth phenotypes than the Not4 deletion and, in particular, was not sensitive to media affecting translation like the deletion of Not4. 2) Protein aggregates accumulated in cells when Not4 was deleted, but much less so when Ccr4 or Caf1 were deleted. 3) Polyubiquitinated proteins accumulated in *not4Δ*, but not in *ccr4Δ* or *caf1Δ* cells. 4) The decay of the proteasomal substrate CPY* in *ccr4Δ* or *caf1Δ* cells was similar to wild-type cells. In contrast, CPY* was stabilized when Not4 was deleted. 5) Finally, proteasome integrity was altered in *not4Δ*, but not in *ccr4Δ* cells.

### Ccr4-Not Complex has Two Different Modules Acting in Quality Control

Many studies have indicated that the Ccr4-Not complex is composed of distinct functional modules. If we consider the 2 enzymes of the complex, microarray analyses revealed that genes deregulated in the ligase *not4Δ* mutant only marginally overlapped with genes deregulated in the deadenylase *ccr4Δ* mutant [76]. In humans Not4 was found outside of the Ccr4-Not complex [21], indicating that some functions of Not4 may not even require its association with the rest of the complex. Nevertheless it has to be considered that, within the Ccr4-Not complex, the E3 ligase might play a role in regulating the deadenylation activity provided by Ccr4. No evidence of this regulatory function is available. In fact there are several indications that the Not4 E3 ligase module does not regulate the deadenylase module: the deletion of Not4 does not dramatically change the composition of the Ccr4-Not complex; Caf1 and Ccr4 still interact with Not1 [76]; mostly normal deadenylase activity occurs *in vivo* in the *not4Δ* mutant [16], and deadenylation of reporter mRNAs was only slightly decreased [30]. One exception is a report, which describes that the accumulation of aberrant translation products in cells lacking Not4 correlates with a slight increase of the related mRNAs [40]. This raised the possibility that Not4 might be necessary to activate the deadenylase module in the context of co-translational quality control, and that this may explain fully that aggregated proteins accumulate in cells lacking Not4.

To address this possibility, we compared the phenotypes of cells lacking one enzyme or the other enzyme. In particular, we wanted to determine whether the phenotypes, which are associated with the loss of Not4 and connected to protein quality control, could be recapitulated by the loss of the deadenylase. This is what one would expect, if the phenotypes in *not4Δ* were due to the lack of activation of the deadenylase. To investigate consequences that are strictly due to loss of deadenylation in yeast, *ccr4Δ* is a better mutant to study than *caf1Δ*. Indeed, in yeast, although Caf1 like Ccr4 can deadenylate substrates *in vitro* [9], [77], [78], only Ccr4 plays a catalytic role *in vivo*, as mentioned above. Caf1 is nevertheless indispensable for deadenylation activity *in vivo* [16], because it functions to bridge Ccr4 to Not1 [18]. However, it seems to play additional roles in the structure of the Ccr4-Not complex [76] and cells lacking Caf1 have more severe phenotypes than cells lacking Ccr4 only.

The deletion of Ccr4 did not have the complete set of phenotypes connected to protein quality control observed in the absence of Not4. It did not impact on proteasome integrity, and it did not result in stabilization of proteasomal substrates, nor did it lead to accumulation of aggregated proteins. Thus, it is highly unlikely that defective deadenylation explains the accumulation of aberrant and aggregated proteins in *not4Δ*.

In contrast to *ccr4Δ*, the Caf1 deletion revealed many phenotypes similar to the deletion of Not4 and not shared by the deletion of Ccr4. The *caf1Δ* mutant was sensitive to HygB and to CHX, and displayed proteasome defects. This might be due to the fact that Caf1 plays structural roles within the Ccr4-Not complex unlike Ccr4, and its absence might impinge on Not4 function. However, *caf1Δ* was not as sensitive to AZC as *not4Δ,* nor was it temperature sensitive, demonstrating that not all functions of Not4 are compromised in *caf1Δ*.

### Not4 Contributes to Protein Quality Control

What then is the role of Not4 in protein quality control and why do aggregated proteins accumulate in the absence of Not4? We show that a proteasomal substrate, CPY*, fails to be degraded in *not4Δ*, compatible with the defective functional integrity of the proteasome in *not4Δ* that we have previously reported. However, in addition, we show that when cells are exposed to proteotoxic shock with a mistranslating agent, AZC, or when temperature is increased and translation stalls, the presence of Not4 in polysomes increases, indicating that Not4 is needed where co-translational responses take place. And indeed, the deletion of Not4 leads to sensitivity of the cells to growth under conditions in which translation is compromised (this article and [35], [75]): HygB affects translational fidelity and increases read-through of stop codons [66]. AZC induces protein misfolding and proteotoxic stress [67], [68]. CHX is a translation inhibitor. All these agents lead to appearance of protein quality control substrates. Sensitive growth phenotypes in the presence of these agents support an important physiological role for Not4 during translation. Consistently, we show that expression from no-go mRNAs is altered in *not4Δ*, indicating that Not4 is important for the co-translational regulation of no-go mRNAs. Aggregates found in *not4Δ* cells contain newly synthesized and polyubiquitinated proteins, supporting the idea that Not4 functions in quality control of *de novo* synthesized proteins. Finally, in good agreement with the idea that both Ltn1 and Not4 are required for co-translation quality control, is the observation that double mutant *not4Δ ltn1Δ* displays a synthetic slow growth phenotype (Fig. S4).

An important question is the co-translational role played by Not4. It was proposed that no-go mRNA translation arrest was accompanied by Not4-dependent ubiquitination and proteasomal degradation of aberrant products [40], but later studies revealed that ubiquitination of arrested proteins mainly occurred by the E3 ligase Ltn1 [41], [79]. Our own previous work showed that Not4 is important for proteasome assembly [35]. In this study we have comparatively analyzed the levels of translationally-arrested proteins and read-through full-length proteins in wild-type, *not4Δ, ccr4Δ*, *caf1Δ*, and *ltn1Δ* cells from no-go mRNAs. We observed that, while in the absence of Ltn1 translationally-arrested proteins accumulated and were less ubiquitinated, as described previously [41], in *not4Δ* they were ubiquitinated to a greater extent. This phenotype was shared by *caf1Δ*, and, hence, may be indicative of defective proteasome activity detected in both *caf1Δ* and *not4Δ*. It is also possible that Not4 and Caf1 limit Ltn1 activity. We also observed that the total level of protein produced from constructs with a stalling amino-acid basic stretch, but not without, was reduced specifically in *not4Δ*. This indicates that Not4 plays a specific role in preserving translation efficiency or mRNA levels from the constructs that lead to translational arrest. An alternative possibility could be that the proteins produced are less stable in the absence of Not4, especially since for one construct, K12, the arrested products were more ubiquitinated. However, this seems unlikely because clearance of proteins by the proteasome in *not4Δ* is less efficient, as indicated by stabilization of CPY*, and, moreover, in *caf1Δ* the K12 arrest products are also more ubiquitinated and yet they accumulate as in wild-type cells.

Bengtson and Joazeiro reported, like us, a reduction of full-length protein product from their no-go K12 constructs in *not4Δ* compared to wild type [41]. However, they discarded this observation as not significant, because they saw a similar decrease from the construct without a stalling sequence [41]. We have carefully looked at this point in our study and did not see the decrease in K0 (Fig. 7). In fact, Dimitrova et al. also observed a reduction of full-length product from their no-go constructs [40]. They did not discuss this at all, because in contrast to us, they saw an increased accumulation of arrested protein from the K12, and even more R12 constructs, in *not4Δ*, and they focused their discussion on this accumulation. This is where our results differ from those of Dimitrova et al., despite the fact that we used the same constructs but in a different strain background (they used W303 and we used BY4741). Bengtson and Joazeiro, who used BY4741 strain background, like us, did not observe increased levels of K12-induced translationally-arrested products in the absence of Not4 only, but they did see such an increase if Ltn1 was deleted.

To understand the inconsistencies and similarities between the results of the 2 previous studies and our current work, it is important to mention that W303 background has sequence differences in genes compared to S288C, from which BY4741 was derived, and these are, in particular, in many stress resistance factors (as explained in the [36]). The deletion of Ltn1 can also be sensed as a stressful situation for the cell. Hence, it could be that translationally-arrested proteins increase in the absence of Not4 upon stress (that could be in W303 background), but decrease in the absence of stress. In such a model, Not4 acts as a switch important to preserve the proteome: in the absence of stress and presence of Ltn1, if translation of an mRNA momentarily stalls, Not4 acts initially to preserve production of full-length protein: it increases translation and/or represses deadenylation. Both functions could be though regulation of the Ccr4/Caf1 module of the Ccr4-Not complex, but could also involve other proteins such as the Dhh1 DEAD box RNA helicase (discussed in [36]). Not4 might also moderate Ltn1 function or have a positive impact on the deubiquitination activity of the proteasome RP, to give a chance for the stalled protein not to be degraded and to be translated into full-length protein. In contrast, upon stress, or if Ltn1 is deleted, Not4 is important to mobilize the deadenylase module of the Ccr4-Not complex to repress translation and/or induce mRNA degradation. Hence, if Not4 is deleted, stalled protein accumulates, and the level of polyubiquitination of this protein will depend upon the presence or not of Ltn1.

In conclusion, in this work we have shown that Not4 is important for cellular protein quality control first, because it is globally important for appropriate clearance of aberrant proteins, because it is important for functional integrity of the proteasome, but also through its function as an E3 ligase, that does not affect proteasome function. Additionally, Not4 is important during translation where it acts as a switch to promote or inhibit production of proteins from stalled mRNAs depending upon the cellular conditions. Determining how the Not4 switch is regulated and exactly operates are obviously now exciting questions to tackle.

## Supporting Information

Figure S1 **Growth phenotypes of the E3 ligases mutants.** The indicated strains were grown to exponential phase and diluted to the same OD_600_ of 0.5. 10-fold serial dilutions were spotted on the YPD plates containing, when indicated, HygB 0.1 mg/ml or CHX 0.05 µg/ml; and left to grow for 6 days (except 16°C) or for 21 days (16°C).
(TIF)

Figure S2 CPY*-HA mRNA and protein levels in wild-type, ***not4Δ, ccr4Δ*** **, and** ***caf1Δ*** **cells. A.** CPY*-HA protein levels in wild-type, *not4Δ, ccr4Δ*, and *caf1Δ* cells. CPY*-HA was expressed from an episome under control of copper dependent promoter in wild-type (WT), *not4Δ, ccr4Δ,* and *caf1Δ* cells. Cells were exponentially grown in the constant presence of 0.1 mM of CuSO_4_ and collected at OD_600_ of 1.0. Different amount of the cells (0.5 OD units (lane 1), 0.125 OD units (lane 2) and 0.05 OD units (lane 3)) were analyzed by SDS-PAGE and western blot with antibodies against HA, to see CPY*-HA levels, and against Egd2 as a loading control. **B.** CPY*-HA mRNA levels in wild-type, *not4Δ, ccr4Δ*, and *caf1Δ* cells. Cells were grown as described in A. 50 OD units of the cultures were collected. Pellets were resuspend in 400 µl of acid phenol and 400 µl of TES buffer (10 mM Tris-HCl pH 7.5, 10 mM EDTA, 0.5% SDS) and incubated at 65°C for 10 min. Samples were chilled on ice for 5 min and spun at 4°C for 10 min. Aqueous phase was extracted with 400 µl of acid phenol and then with chloroform. Finally, RNA was collected by ethanol/sodium acetate precipitation. 4 µg of the RNA were treated with DNAse (Promega) and than reverse transcribed with M-MLV RT (Promega) according to the manufacturer’s instructions and using oligo d(T) primers (Qiagen). SYBR green based quantitative RT-PCR was performed using BioRad cycler. *ACT1* was used as a housekeeping gene and CPY*-HA signals were normalized on *ACT1* level. The ratio CPY*-HA/ACT1 in wild type was normalized to 1. Primers used for analysis: The forward primer: 5′-TCCCCGGGTTAATTAACATC-3′ and reverse primer: 5′-TCGCTTATTTAGAAGTGGCG-3′ amplify 149 bp fragment of HA tag of CPY*-HA gene. The forward primer: 5′-TTGTCCGTGACATCAAGGAA-3′ and reverse primer: 5′-ACCCAAAACAGAAGGATGGA-3′ amplify 182 bp fragment of *ACT1* gene.
(TIF)

Figure S3 **Polysome profiles from wild-type,** ***not4Δ*** **, and** ***ccr4Δ*** **cells.** Extracts from wild-type (black), *not4Δ* (red), and *ccr4Δ* (blue) cells, containing 3 mg of total proteins, were subjected to 7–47% sucrose gradient centrifugation and analyzed by UV reading at 254 nm (left). Profiles were superposed (right). The positions of 40S, 60S, 80S, and polysomes are indicated.
(TIF)

Figure S4 **Double mutant** ***ltn1Δ not4Δ*** **grows slowly compare to single** ***ltn1Δ*** **or** ***not4Δ*** **mutants.**
(TIF)
