# Supplementary figures and images for: The Not4 E3 Ligase and CCR4 Deadenylase Play Distinct Roles in Protein Quality Control
Source: PLoS One. 2014 Jan 17;9(1):e86218. doi: 10.1371/journal.pone.0086218 (PMC3895043; doi:10.1371/journal.pone.0086218)

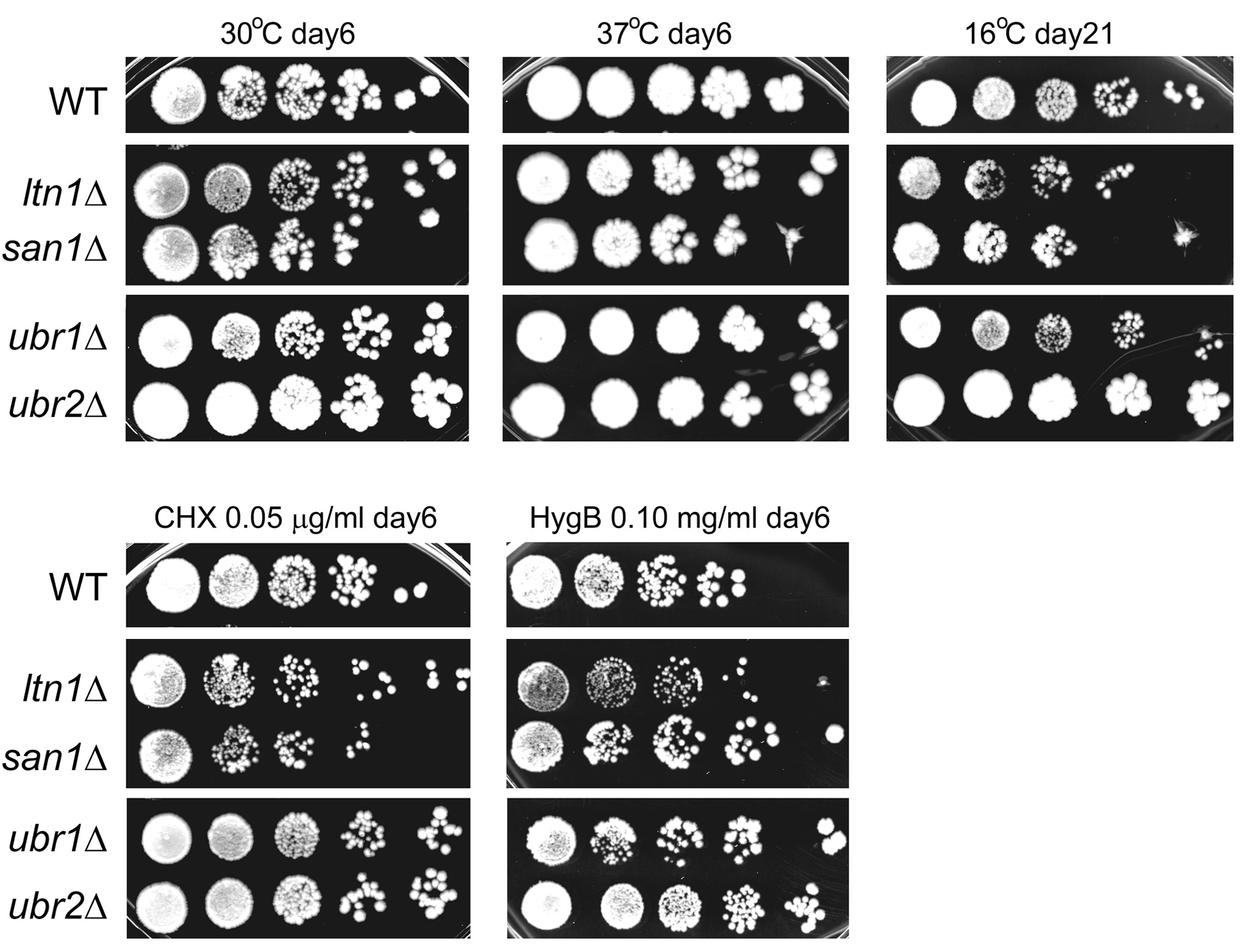

Supplement: Figure S1 — Growth phenotypes of the E3 ligases mutants. The indicated strains were grown to exponential phase and diluted to the same OD600 of 0.5. 10-fold serial dilutions were spotted on the YPD plates containing, when indicated, HygB 0.1 mg/ml or CHX 0.05 µg/ml; and left to grow for 6 days (except 16°C) or for 21 days (16°C). (TIF) [file pone.0086218.s001.tif]

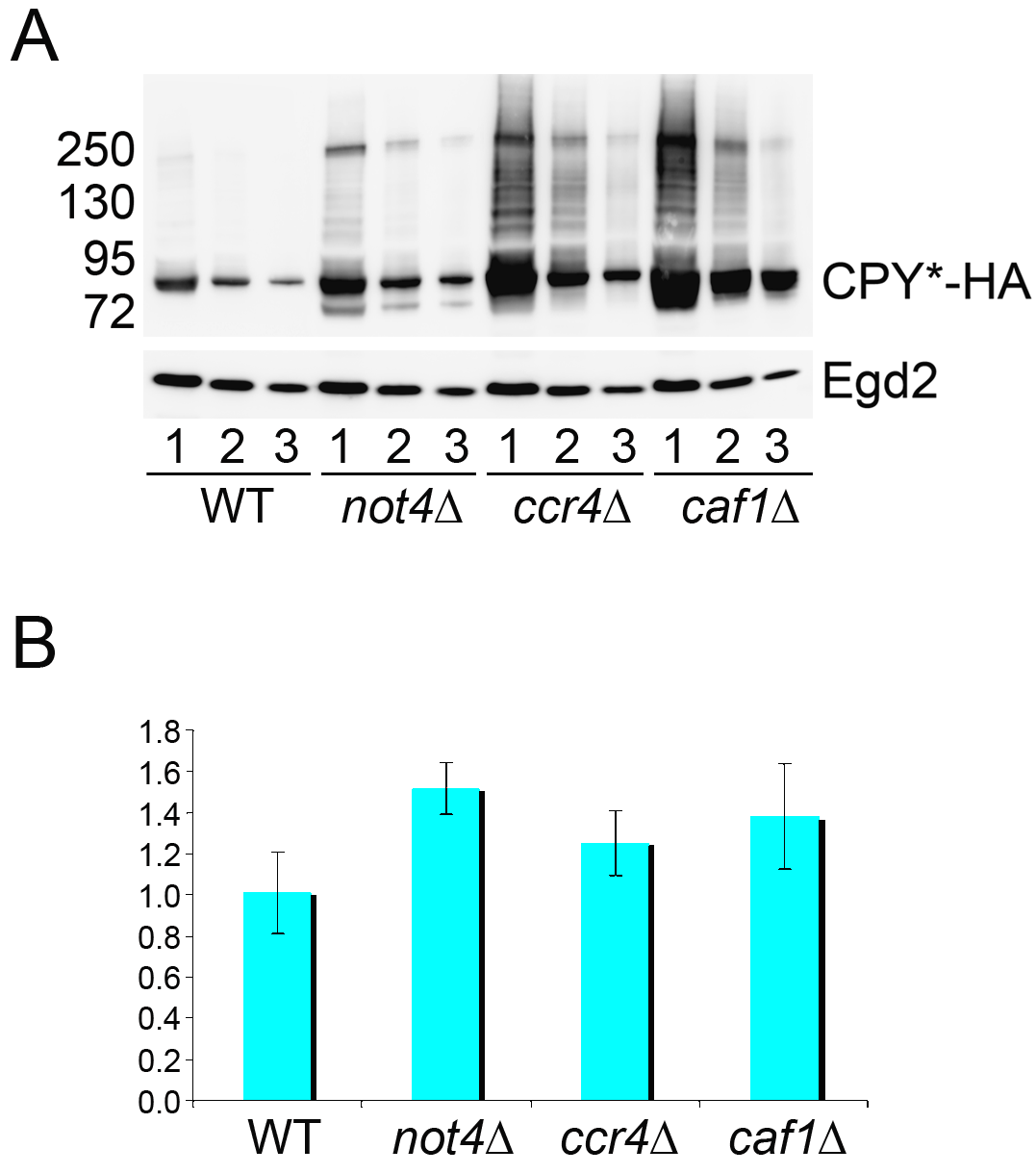

Supplement: Figure S2 — CPY*-HA mRNA and protein levels in wild-type, not4Δ, ccr4Δ , and caf1Δ cells. A. CPY*-HA protein levels in wild-type, not4Δ, ccr4Δ, and caf1Δ cells. CPY*-HA was expressed from an episome under control of copper dependent promoter in wild-type (WT), not4Δ, ccr4Δ, and caf1Δ cells. Cells were exponentially grown in the constant presence of 0.1 mM of CuSO4 and collected at OD600 of 1.0. Different amount of the cells (0.5 OD units (lane 1), 0.125 OD units (lane 2) and 0.05 OD units (lane 3)) were analyzed by SDS-PAGE and western blot with antibodies against HA, to see CPY*-HA levels, and against Egd2 as a loading control. B. CPY*-HA mRNA levels in wild-type, not4Δ, ccr4Δ, and caf1Δ cells. Cells were grown as described in A. 50 OD units of the cultures were collected. Pellets were resuspend in 400 µl of acid phenol and 400 µl of TES buffer (10 mM Tris-HCl pH 7.5, 10 mM EDTA, 0.5% SDS) and incubated at 65°C for 10 min. Samples were chilled on ice for 5 min and spun at 4°C for 10 min. Aqueous phase was extracted with 400 µl of acid phenol and then with chloroform. Finally, RNA was collected by ethanol/sodium acetate precipitation. 4 µg of the RNA were treated with DNAse (Promega) and than reverse transcribed with M-MLV RT (Promega) according to the manufacturer’s instructions and using oligo d(T) primers (Qiagen). SYBR green based quantitative RT-PCR was performed using BioRad cycler. ACT1 was used as a housekeeping gene and CPY*-HA signals were normalized on ACT1 level. The ratio CPY*-HA/ACT1 in wild type was normalized to 1. Primers used for analysis: The forward primer: 5′-TCCCCGGGTTAATTAACATC-3′ and reverse primer: 5′-TCGCTTATTTAGAAGTGGCG-3′ amplify 149 bp fragment of HA tag of CPY*-HA gene. The forward primer: 5′-TTGTCCGTGACATCAAGGAA-3′ and reverse primer: 5′-ACCCAAAACAGAAGGATGGA-3′ amplify 182 bp fragment of ACT1 gene. (TIF) [file pone.0086218.s002.tif]

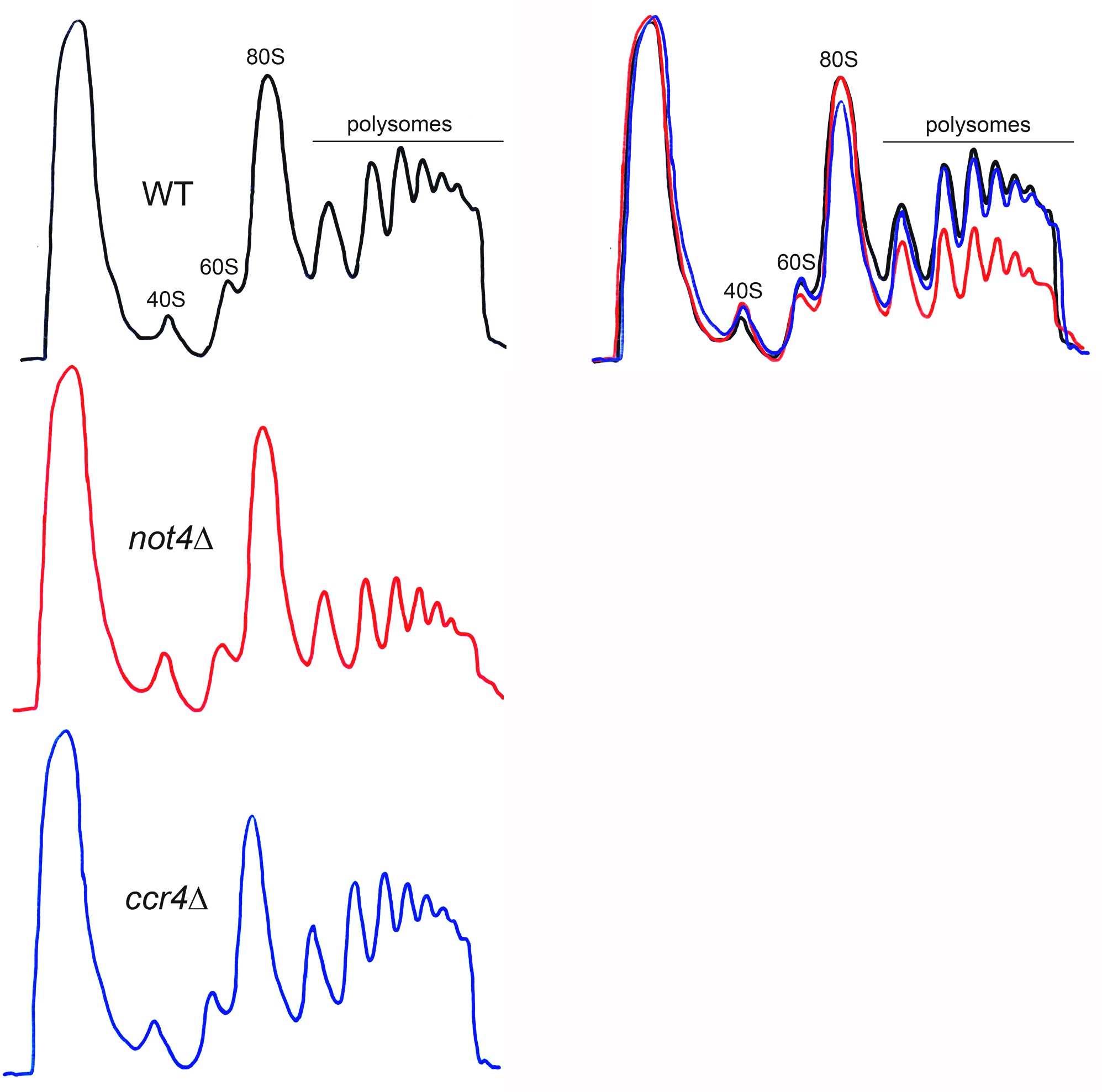

Supplement: Figure S3 — Polysome profiles from wild-type, not4Δ , and ccr4Δ cells. Extracts from wild-type (black), not4Δ (red), and ccr4Δ (blue) cells, containing 3 mg of total proteins, were subjected to 7–47% sucrose gradient centrifugation and analyzed by UV reading at 254 nm (left). Profiles were superposed (right). The positions of 40S, 60S, 80S, and polysomes are indicated. (TIF) [file pone.0086218.s003.tif]

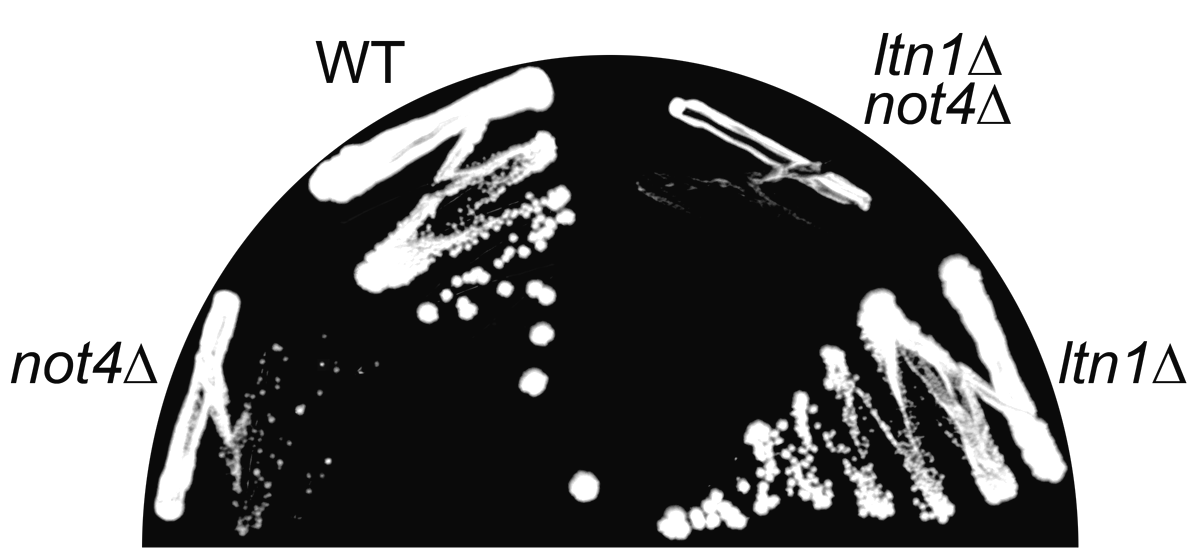

Supplement: Figure S4 — Double mutant ltn1Δ not4Δ grows slowly compare to single ltn1Δ or not4Δ mutants. (TIF) [file pone.0086218.s004.tif]
